# Serum Bilirubin Levels in Overweight and Obese Individuals: The Importance of Anti-Inflammatory and Antioxidant Responses

**DOI:** 10.3390/antiox10091352

**Published:** 2021-08-26

**Authors:** Lovro Žiberna, Zala Jenko-Pražnikar, Ana Petelin

**Affiliations:** 1 Institute of Pharmacology and Experimental Toxicology, Faculty of Medicine, University of Ljubljana, SI-1000 Ljubljana, Slovenia; lovro.ziberna@mf.uni-lj.si; 2 Faculty of Health Sciences, University of Primorska, SI-6310 Izola, Slovenia; zala.praznikar@upr.si

**Keywords:** adipokines, antioxidant, anti-inflammatory, bilirubin, obesity, overweight

## Abstract

Obesity is a chronic condition involving low-grade inflammation and increased oxidative stress; thus, obese and overweight people have lower values of serum bilirubin. Essentially, bilirubin is a potent endogenous antioxidant molecule with anti-inflammatory, immunomodulatory, antithrombotic, and endocrine properties. This review paper presents the interplay between obesity-related pathological processes and bilirubin, with a focus on adipose tissue and adipokines. We discuss potential strategies to mildly increase serum bilirubin levels in obese patients as an adjunctive therapeutic approach.

## 1. Introduction

Bilirubin, the end product of heme metabolism, is a potent endogenous antioxidant with anti-inflammatory, immunomodulatory, antithrombotic, and endocrine properties [1,2,3]. Serum bilirubin concentrations depend on the complex interactions between bilirubin production, consumption (depending on oxidative stress and inflammation), metabolism, and elimination. Importantly, numerous studies have shown that serum bilirubin levels are inversely associated with obesity, metabolic syndrome (MetS), type 2 diabetes mellitus (T2DM), and other oxidative-stress-mediated diseases, including atherosclerosis [2,4,5]. Moreover, serum bilirubin levels were recently proposed as a potential pre-disease biomarker for developing metabolic syndrome in asymptomatic middle-aged individuals [6]. 

Obesity and overweight are considered pathological states of chronic low-grade inflammation with increased oxidative stress and altered endocrine signaling; therefore, these conditions result in lowered serum bilirubin levels [6]; on the other hand, reducing body weight leads to increased serum bilirubin levels [7,8]. Importantly, mild hyperbilirubinemia is associated with health benefits in overweight and obese individuals, as well as with lower adiposity [9,10]. This review addresses the current knowledge on how overweight and obesity affect bilirubin levels, along with the potential intervention strategies to modulate systemic bilirubin levels to alleviate the obesity-related negative effects on health.

## 2. Inflammation in Obesity

Obesity is the accumulation of excessive fat that harms health. Indeed, obesity and overweight are associated with several dysmetabolic conditions, including T2DM, non-alcoholic fatty liver diseases, cardiovascular diseases (CVDs), cancer, and neurodegenerative disorders, among others. Recently, the understanding of adipose tissue has undergone radical changes. Adipose tissue has been recognized as a heterogeneous tissue; indeed, it is composed of several cell types, including preadipocytes, mature adipocytes, fibroblasts, dendritic cells, mast cells, T-cells, endothelial cells, and macrophages [11]. Importantly, obesity is linked to a state of chronic low-grade inflammation, mainly due to proinflammatory cytokine secretion, macrophage infiltration, and disrupted function of tissue involved in glucose homeostasis [12]. Additionally, obesity is also associated with a significant increase in macrophage number [13,14], which also contributes to the maintenance of the low-grade chronic inflammation state linked to obesity [15]. Macrophages are increased in adipose tissue during obesity due to several factors, free fatty acids, cholesterol, and lipopolysaccharide [16]. Macrophages can be classified, based on their surface expression or their cytokine or chemokine expression, into two main populations. M1 macrophages are associated with a proinflammatory profile and secrete proinflammatory cytokines (tumor necrosis factor α (TNF-α), interleukin 1ß (IL-1ß), and interleukin 6 (IL-6)), whereas M2 macrophages are associated with tissue remodeling and inflammation resolution and secrete anti-inflammatory cytokines (IL-10, IL-1) [17,18]. Moreover, treating macrophages with a mix of glucose, palmitate, and insulin generates a unique macrophage proinflammatory phenotype that is different from M1 and secretes proinflammatory cytokines (TNF-α, IL-1ß), while the secretion depends on peroxisome-proliferator-activated receptor gamma (PPAR-γ) and p62 expression [19]. The mechanisms by which inflammation increases during obesity are still not fully understood. First, increased proinflammatory cytokine secretion contributes to insulin resistance and other complications related to obesity. Second, obesity not only promotes the infiltration of macrophages but also induces a shift in macrophage balance toward the M1 phenotype [17]; however, the adipose tissue is not the sole site of inflammation. In contrast, obesity-related inflammation occurs in many other tissues, such as the liver, muscle, hypothalamus, pancreatic islets, and the gut [20].

Adipose tissue was considered as an inert tissue having a primary role in controlling energy homeostasis. Additionally, it is now recognized that adipose tissue exhibits endocrine-regulatory properties and releases a cluster of bioactive substances, among them hormones and adipokines with pleiotropic functions [11,21,22,23]. Adipokines comprise, among others, classical cytokines (TNFα, IL-6) and chemokines (IL-8, monocyte chemoattractant protein 1 (MCP-1), macrophage-inflammatory protein-1α, macrophage-inflammatory protein-2α, stromal-cell-derived factor-1), vasoactive and coagulation factors, regulators of lipoprotein metabolism, and proteins more specifically secreted by the adipocytes, such as leptin and adiponectin [24]. Adipocytes have been recognized as important sources of MCP-1, which was the first discovered human ß-chemokine and is a recognized marker of adipose tissue dysfunction in obesity and T2DM [25]. An early study determined a link between obesity and inflammation and showed that adipose tissue synthesizes and releases the proinflammatory cytokine TNF-α [26]. Based on these findings, it was suggested that adipose tissue plays an important immune role and might be a major source of proinflammatory mediators, which initiate the development of low-grade chronic inflammation. Indeed, excess adipose tissue leads to high levels of proinflammatory cytokines TNF-α and IL-6 and a sensitive marker of inflammation C-reactive protein (CRP) in circulating blood. TNF-α is synthesized as a 26 kDa transmembrane protein. It has been shown that macrophages from the stromal vascular fraction are also the source of adipose-derived TNF-α and that its increased levels in obesity are due to the increased infiltration of adipose tissue with M1 macrophages [13]. Several studies have demonstrated that TNF-α impairs insulin signaling in hepatocytes and adipose tissue [27,28]. Moreover, IL-6 is a cytokine produced by many different cells. Approximately one-third of the IL-6 detected in plasma is attributed to the production from white adipose tissue [29]. In adipocytes and hepatocytes, IL-6 has been demonstrated to impair insulin-induced insulin receptor and insulin receptor substrate 1 phosphorylation [30,31]. Furthermore, it has been shown that IL-6 stimulates hepatocytes to produce and secrete CRP, indicating a state of inflammation [32]. CRP is a sensitive marker of inflammation, which is synthesized and secreted mainly by the liver [32], the serum concentrations of which are higher among obese subjects [33,34]. Adiponectin is one of the most abundant adipokines produced by adipocytes and is involved in glucose and lipid metabolism [35]. In adipose tissue, adiponectin exerts an anti-inflammatory function by reducing macrophage infiltration and inhibiting the local production of numerous proinflammatory cytokines [36]. Leptin, which is almost exclusively secreted from adipocytes, controls food intake and energy expenditure and has atherogenic and growth properties [37]. Since the discovery of leptin, the number of adipokines has notably increased in the last years with new molecules such as omentin-1, chemerin, resistin, visfatin, apelin, adipocyte fatty acid-binding protein (A-FABP), retinol-binding protein 4 (RBP4), among others [38]. Visfatin, originally identified as a pre-B-cell colony-enhancing factor (PBEF), is expressed in many cells and tissues act as a cytokine with immune regulatory action and as nicotinamide phosphoribosyltransferase (Nampt), an enzyme involved in the NAD^+^ salvage pathway [39]. 

## 3. Anti-Inflammatory Activity of Bilirubin 

Bilirubin has evident anti-inflammatory activity, and indeed several studies have shown an inverse relationship between serum bilirubin levels and CRP in overweight or diabetic subjects [5,8,40,41]. Since low-grade chronic inflammation plays an important role in adipose tissue, the liver, the aorta, the kidneys, and pancreatic cells, the anti-inflammatory and antioxidative effects of bilirubin may likely contribute to the protective effect on vascular damage [1]. Recently, adipokines were studied along with serum bilirubin levels in normal and overweight asymptomatic adults. Importantly, there was an inverse relationship between serum bilirubin levels and proinflammatory cytokines (TNF-α, IL-6, visfatin, and CRP) and a positive relationship between serum bilirubin levels and adiponectin [5,42].

Moreover, recently biliverdin treatment reduced the expression levels of M1 macrophage markers in adipose tissues induced by high-fat diet feeding mice. This indicates that bilirubin may improve high-fat-diet-induced insulin resistance by reducing chronic inflammation in adipose tissue [10]. 

Additionally, adiponectin exerts anti-inflammatory and antiatherogenic properties via its ability to stimulate vascular endothelial nitric oxide (NO) production [43]. Equivalently, more recent studies have shown the role of bilirubin in the activation of Akt and endothelial nitric oxide synthase leading to the synthesis of NO, which can also improve endothelial cell function and insulin resistance [44]. 

To address the potency of bilirubin responses, in several animal models of endotoxemia, septicemia, and ischemia–reperfusion injury, bilirubin exhibited significant anti-inflammatory activity via mechanisms such as inhibiting the expression of adhesion molecules, suppressing the infiltration of inflammatory cells and reducing the levels of proinflammatory cytokines [45]. In another study, bilirubin also suppressed T cell proliferation and activation [46]. Overall, bilirubin has complex immunosuppressive effects [47]. A recent study also identified the neutrophil-to-lymphocyte ratio in blood as a variable that is negatively associated with total bilirubin levels [48].

Further studies are warranted to better elucidate bilirubin’s mechanisms of action on adipocytes and to confirm the correlation between serum bilirubin and adipokines.

Table 1 presents adipokines and inflammatory markers that are altered in obese and overweight individuals and the relationships with serum bilirubin levels (if known).

## 4. Oxidative Stress in Adipose Tissue Due to Obesity

In addition to an increased proinflammatory response in adipose tissue due to overweight and obesity, excessive production of reactive oxygen species (ROS), decreased antioxidant activity, and higher oxidative stress, especially in white adipose tissue, are also observed in overweight and obese individuals [52,53,54]. 

In overweight and obese individuals, oxidative stress in adipose tissue is mainly induced by the catalytic activity of the enzyme nicotinamide adenine dinucleotide phosphate (NADPH) oxidase (NOX) or by dysfunctional mitochondrial oxidative phosphorylation [52,55]. Electron transfer through the electron transfer chain or from NADPH to oxygen generates superoxide anions, which are the primary ROS species that are further converted to H_2_O_2_; however, ROS include chemically diverse compounds (nitric oxide, peroxynitrite, hypochlorous acid, singlet oxygen, hydroxyl radical) with diverse physiological and pathological effects.

Several studies have shown that NOX-derived ROS production is associated with the early stages of obesity, while mitochondrial-derived ROS production is associated with the late stages of obesity [55]. NOX is a plasma-membrane-bound enzyme involved in the transfer of electrons from NADPH to oxygen and is the main generator of ROS in adipocytes [56]. In obese mice, mRNA expression levels of NOX subunits were increased only in adipose tissue, which was associated with increased ROS production [53], while adipose-specific deletion of NOX4 attenuated adipose tissue inflammation and early onset of insulin resistance in diet-induced obese mice [57]. Moreover, in cultured 3T3-L1 adipocytes, high levels of free fatty acids (FFAs) and glucose increased ROS production via NOX activation [53,58], whereas treatment with NOX inhibitors or silencing of NOX4 ameliorated this effect by reducing ROS generation [53,58].

In overweight and obese individuals, oxidative stress in adipose tissue is also induced by dysfunctional mitochondrial oxidative phosphorylation. Conditions that favor mitochondrial superoxide production include a reduction in electron carrier pools associated with the mitochondrial respiratory chain (NADH, flavins, ubiquinone), high proton motive force, and increased oxygen consumption within mitochondria [59]. Overeating delivers excess electrons to the respiratory chain, while lack of physical activity and low ATP demand promote a high proton motive force at a low respiratory rate, leading to mitochondrial superoxide formation and oxidative stress. In a morbidly obese state, adipocytes utilize FFAs from triglyceride stores via excessive lipolysis for energy because of glucose deprivation due to insulin resistance [55]. Excess FFAs lead to an excess of electrons in the electron transport chain during oxidative phosphorylation, resulting in their exit and the generation of O_2_^−^ followed by the production of other ROS molecules [60]. Increased β-oxidation also leads to an increased mitochondrial NADH/NAD^+^ ratio, resulting in increased activation of protein kinase C (PKC). Activated PKC then contributes to ROS production by increasing the activity of NADPH oxidase (NOX) [60]. 

Excessive production of mitochondrially derived ROS is also associated with exacerbation of inflammation through the release of inflammatory cytokines and proinflammatory transcription factors such as nuclear factor kappa B (NF-κB) and activator protein-1, which in turn increases ROS production [61]. Moreover, activated PKC induces NF-κB and tumor growth factor-beta activation, establishing a link between oxidative stress and inflammation. The phenomenon of increased proinflammatory response and oxidative stress in obese states contributes significantly to the development of other metabolic complications such as T2DM, cardiovascular disease, and certain types of cancer [62].

## 5. Antioxidant Activity of Bilirubin

Bilirubin is a potent endogenous antioxidant at its physiological concentrations. Bilirubin can scavenge reactive oxygen species by oxidizing itself to biliverdin. Then, biliverdin is recycled back to bilirubin by biliverdin reductase [63]. This cycle enables nanomolar concentrations of bilirubin to protect cells from the 10,000-fold molar excess of oxidants when both substances are added exogenously to cell culture [64]. 

In addition to the direct ROS scavenging activity, bilirubin can further decrease oxidative burden in the intracellular compartment by inhibiting NADPH oxidase complexes, which are the major source of oxidative stress in adipocytes [65,66]. Moreover, activated macrophages in hypertrophic adipose tissue express NADPH oxidase and further increase oxidative stress. Altogether, the redox imbalance leads to adipocyte dysfunction in obesity [56]. Importantly, bilirubin, via its antioxidant activity, can prevent adipocyte hypertrophy via its hypothalamic effect and can improve the function of adipocytes that have already hypertrophied [9,56].

Obesity and MetS also impair vascular endothelial function; however, bilirubin also acts as an intracellular antioxidant in the endothelia of both arterial and venous systems, with EC_50_ values in the nanomolar range [67].

## 6. Bilirubin as a Signaling Molecule Involved in Energy Homeostasis

In addition to bilirubin’s known role as an antioxidant and anti-inflammatory molecule, it is now recognized that unconjugated bilirubin is also a potent endogenous activator of several ligand-activated transcriptional factors crucially involved in metabolic homeostasis, including peroxisome proliferator-activated receptor alpha (PPAR-α), aryl hydrocarbon receptor, constitutive androsterone receptor (CAR), liver X receptors (LXRs), and pregnane X receptor (PXR) [68].

PPARs, a class of nuclear receptors, function as fatty acid and eicosanoid sensors that regulate multiple pathways involved in lipid and glucose metabolism, as well as overall energy metabolism, playing an important role in the pathogenesis of obesity and other metabolic diseases [69]. Interestingly, there are structural similarities between bilirubin and known PPARα ligands [70]. Furthermore, bilirubin explicitly docks to the PPAR-α ligand-binding domain to regulate transcriptional responses. PPAR-α is highly active in the liver, adipose tissue, kidney, heart, and muscle tissue [71], where it regulates the adaptive response to prolonged fasting by controling the process of ketogenesis, fatty acid transport, fatty acid binding, fatty acid activation, and mitochondrial fatty acid-β oxidation [72]. Genomic studies have shown that PPAR-α, as a master regulator of lipid metabolism, has several target genes; classic genes include acyl-CoA oxidase, thiolase, fatty acid transport protein, carnitine palmitoyltransferase I, and peroxisome proliferator-activated receptor-gamma coactivator 1-alpha [72]. Recent studies on bilirubin-induced transcriptome responses showed that it selectively acts via PPAR-α, inducing gene transcription that activates mitochondrial function and β-oxidation to utilize fats for energy and reduces body weight [70,73,74,75]. In addition, hyperbilirubinemia in mice on a high-fat diet increased the mitochondrial number and hyperphosphorylation of PPAR-α compared to controls, thereby indicating that bilirubin is a metabolic hormone that controls white adipose tissue expansion and reduces hypertrophy and glucose intolerance [76]. Furthermore, bilirubin nanoparticles induce hepatic fat utilization, increase plasma ketones, and reduce hepatic steatosis [77].

In addition to bilirubin’s direct action as a PPAR-α ligand, bilirubin acts via FABP1, CAR, and LXRs [68]. CAR has recently been identified as a therapeutic target for obesity and its associated metabolic disorders [78,79]. LXRs are sterol sensors that mainly regulate cholesterol, fatty acids, and glucose homeostasis, and may inhibit the development of atherosclerosis but promote lipogenesis in the liver [80]. Bilirubin acts as a ligand transactivator of CAR and PXR to increase the expression of target genes responsible for its disposal [81], and also to increase the expression of genes involved in energy metabolism [82]. On the other hand, LXR is under negative control of bilirubin and systemic bilirubin application was shown to attenuate dyslipidemia in diabetic rats [83].

**Figure 1:**
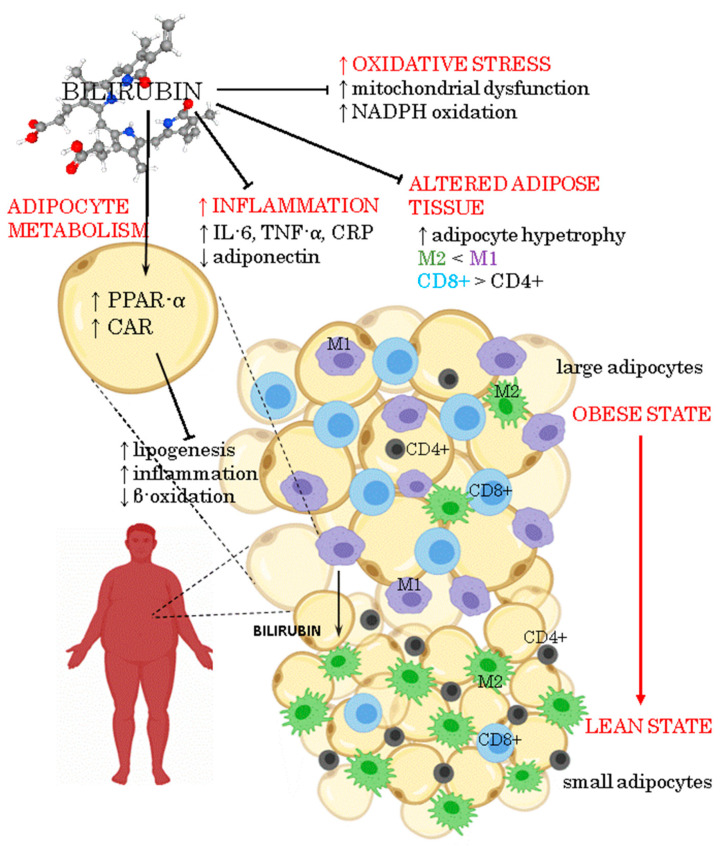
Pleiotropic effects of bilirubin on obesity.

Figure 1 illustrates the anti-inflammatory and antioxidant actions of bilirubin and its properties as a signalling molecule in adipose tissue.

## 7. Potential Interventions to Modulate Serum Bilirubin Levels in Obesity

Strategies to mildly increase serum bilirubin levels are important, especially in individuals most at risk. It is important to note that already mild elevation of bilirubin levels can have important clinical benefits. For example, each 0.1 mg/dL (1.7 μM) increase in serum bilirubin levels was associated with a 6% reduction in the risk of peripheral arterial disease [84]. Each micromolar increase in serum bilirubin was associated with an 11% decrease in the odds of developing diabetes [85] and decreased systolic blood pressure by 0.13 mm Hg [86]. Both of these two conditions, diabetes mellitus and hypertension, are correlated with obesity.

Low serum bilirubin levels (hypobilirubinemia) are harmful and can lead to various cardiovascular complications and possibly stroke [87]. Lower bilirubin levels contribute to increased low-density lipoprotein oxidation in obese children and adolescents, predisposing them to increased cardiovascular risk [88]. Indeed, serum bilirubin concentrations were significantly lower in patients with acute myocardial infarction [89]. Moreover, low serum bilirubin levels (<7 μM) are correlated with the significant increase in the relative risk of coronary heart disease [90] and with the risk of overall mortality [91]; therefore, strategies to increase serum bilirubin levels represent an important approach to ameliorate several obesity-mediated or obesity-correlated diseases.

**Figure 2:**
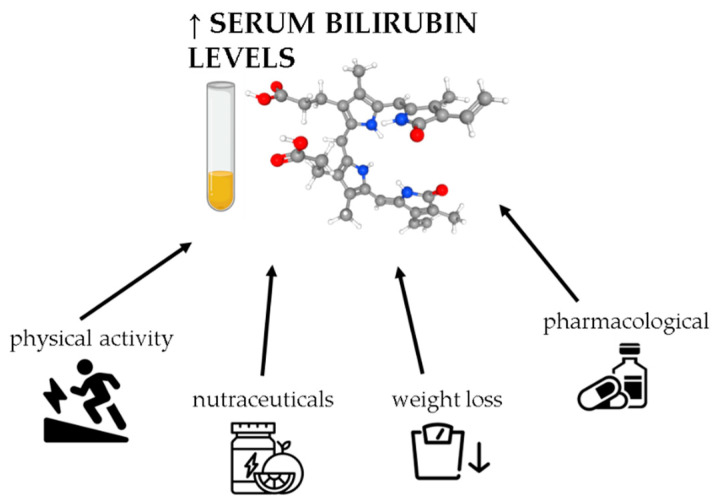
Approaches to modulate serum bilirubin levels in obesity. Bilirubin can be upregulated by different types of interventions, including exercise, diet, pharmacological, and nutraceutical interventions.

Figure 2 illustrates different interventions to modulate serum bilirubin levels in obesity.

### 7.1. Weight Loss

Weight loss is the most obvious approach for treating obesity, as well as in the modulation of serum bilirubin levels. Low caloric intake in obese patients in the period between one and two months increased serum bilirubin levels by 18–45% [92,93]. Similarly, short-term weight loss effectively increased bilirubin levels with an increase in bilirubin as a linear function of weight change [8]. The effect was higher in men than in women; namely, each 1% increase in weight loss was associated with a 0.21 μM increase in serum bilirubin levels in men and a 0.11 μM increase in women [8]. Additionally, there is a direct correlation between BMI and serum bilirubin levels in asymptomatic overweight individuals [6], as well as in obese patients [48].

In addition to weight loss, changes in body composition are also important. Body fat percentage was inversely correlated with bilirubin in obese patients but not in controls [94].

### 7.2. Nutraceutical Interventions

Diet affects serum bilirubin levels. A higher intake of flavonoid-rich fruits and vegetables was significantly associated with higher total serum bilirubin [95], while a higher intake of fried and fast foods was significantly associated with lower bilirubin levels [96,97]. Indeed, we have previously shown that higher intake of total fatty acids is associated with lower serum bilirubin, while higher intake levels of vitamin C and folic acid are associated with higher bilirubin levels [6].

It has been shown that many natural compounds, including curcuminoids, flavonoids, epigallocatechin-3-gallate, genistein, caffeic acid, resveratrol, and natural coumarins, can induce the expression of heme oxygenase-1 (HO-1) [98,99,100]. Indeed, in our previous work, we showed that consumption of buckwheat porridge rich in flavonoids for four weeks resulted in higher serum bilirubin levels in subjects with MetS [101]. In addition to polyphenols, other bioactive natural compounds also exert HO-1 inducing activity. Our group recently showed that peroral application of lyophilized royal jelly significantly increased serum bilirubin compared to placebo after 8 weeks in asymptomatic overweight adults [102]. The bioactive compound was likely 4-hydroperoxy-2-decenoic acid ethyl ester, a royal jelly fatty acid derivative that significantly induced HO-1 expression in cell culture [103].

Moreover, the HO-1 enzyme combines the physiology of the bilirubin and adiponectin axis responses. Oxidative stress and inflammation upregulate the level of inducible HO-1, thereby increasing total bilirubin levels [104]. Furthermore, the HO-1 system is also involved in enhancing adiponectin synthesis and release [105]. Interestingly, HO-1 induction was reported to reduce visceral and subcutaneous obesity in diabetic and obese mice [106]. Likewise, increasing intracellular bilirubin levels via HO-1 induction in adipocytes improved adipocyte function and adipose remodeling by increasing adiponectin levels [107]. In a similar study, HO-1 induction also altered the physical appearance of adipocytes from a few large to many smaller adipocytes [108]. Additionally, the HO-1 induction was found to be beneficial in obesity by inhibiting adipogenesis by preventing the creation of adipose tissue from mesenchymal stem cells [109].

In addition to stimulatory effects on HO-1, many natural bioactive compounds, including epigallocatechin gallate, flavonoids, silymarin, flavonolignans, and various herbal supplements, exhibit inhibitory effects on UDP glucuronosyltransferase family 1 member A1 (UGT1A1) [85,110,111], thereby leading to hyperbilirubinemia. On the other hand, a bilirubin-lowering effect has been observed with the intake of citrus fruits, cruciferous vegetables, onions, garlic, and coffee, probably due to the UGT1A1-inducing activities [85,112].

Another potential dietary approach is to enhance the systemic pool of tetrapyrroles with bilirubin-like substances. In vitro experiments on cell cultures using the edible blue alga Spirulina platensis, which is rich in tetrapyrrolic compounds closely related to bilirubin, led to improved intracellular redox status and antiproliferative effects [113].

### 7.3. Physical Activity

One of the first reports showed that any type of exercise, either habitual or irregular, increased total bilirubin levels compared with the group of individuals who do not exercise [114]. Indeed, intensive regular exercise, as seen in athletes, is correlated with elevated concentrations of total bilirubin [115]. Animal experiments showed that exercise stimulates pathways that raise serum bilirubin levels through alterations in the levels of hepatic enzymes; namely, increasing the expression of biliverdin reductase and consequently increasing bilirubin synthesis, as well as decreasing the expression of UGT1A1, which converts the biologically active unconjugated bilirubin into the non-active conjugated form [116].

The intensity of the exercise is important in increasing the serum bilirubin levels. An intervention study on previously sedentary postmenopausal women showed that only a high dose of exercise training of at least 12 kilocalories per kilogram per week of exercise training at an intensity of 50% of aerobic capacity resulted in a modest elevation of serum bilirubin levels, whereas lower training loads had no effect [117]. Accordingly, asymptomatic overweight and obese middle-aged individuals who had better measured aerobic body capabilities had higher serum bilirubin levels [6].

Some studies have shown no effects of exercise on the modulation of serum bilirubin levels. A study on women with abdominal obesity doing endurance or endurance–strength exercise for 60 min 3 times/week for 3 months did not alter total serum bilirubin levels [118]. Moreover, endurance–strength exercise even decreased indirect bilirubin [118]. Similarly, another study showed that moderate-to-vigorous physical activity increased bilirubin levels only in insulin-resistant individuals but not in insulin-sensitive individuals [119].

### 7.4. Pharmacological Approaches

Diverse pharmacological strategies can modulate serum bilirubin levels via the application of drug compounds that bind to the selected molecular targets, affecting the following bilirubin homeostasis processes: (i) the synthesis of bilirubin (HO-1, biliverdin reductase); (ii) the metabolism of bilirubin UGT1A1, which converts the active unconjugated form of bilirubin into non-active conjugated bilirubin; (iii) the uptake of bilirubin via membrane transporters (bilitranslocase, organic anionic transporter OATP1B1) into the hepatic and other cells; (iv) the distribution of bilirubin throughout the body via serum albumin binding.

To increase the synthesis of bilirubin, several potential drug compounds can increase the level of HO-1, thereby leading to either increases in intracellular and serum bilirubin levels [120], namely nonsteroidal anti-inflammatory drugs such as acetylsalicylic acid and coxibs and hypolipidemic drugs such as statins, fibrates, and niacin [121]. In mouse experiments, atorvastatin and rosuvastatin increased expression of HO-1, consequently increasing serum bilirubin levels and total antioxidant status [122]. Importantly, 60–70% of patients with obesity are dyslipidemic, with statins representing the cornerstone of dyslipidemia treatments [123].

Another approach to mildly elevated bilirubin levels is to reduce bilirubin metabolism by inhibition of UGT1A1. Atazanavir is a drug compound used clinically for treating HIV-infected patients; however, it is also a well-established inhibitor of UGT1A1 leading to increased unconjugated bilirubin levels [124]. Indeed, patients on atazanavir therapy have less myocardial infarction compared with other pharmacotherapy strategies [125]. Atazanavir treatment for 3 days in non-HIV patients with T2DM increased average serum bilirubin levels from 7 to 64 μM, increased total plasma antioxidant capacity, and improved endothelium-dependent vasodilation [126]. Other drugs known to inhibit UGT1A1 are cancer treatment drugs, such as kinase inhibitors and topoisomerase I inhibitors, among others [121]; however, these latter drugs are less suitable for modulating serum bilirubin levels in obese patients due to their serious adverse effects.

There is also a large group of drugs that inhibit the uptake of bilirubin into the liver and other cells. Inhibition of entry into liver cells will decrease bilirubin metabolism, thereby increasing unconjugated bilirubin, which is also the bioactive form. Some drug compounds can act as inhibitors of bilitranslocase, a membrane protein that acts as a transporter of bilirubin from blood to liver cells [127]. Some promising candidate drugs are the antihypertensive drug hydrochlorothiazide and the nonsteroidal anti-inflammatory drug sulindac [127]. In addition to the bilitranslocase, bilirubin can enter cells using OATP1B1 transporter, which interacts with more than 700 drug compounds; some reported candidate drugs are statins, nonsteroidal anti-inflammatory drugs, β-blockers, cyclosporin A, antivirals, sartans, glitazones, and many others [121].

Lastly, several drug compounds compete with bilirubin for albumin binding, displacing unconjugated bilirubin from the binding site on albumin, leading to transient hyperbilirubinemia [128]. For example, ibuprofen replaces bilirubin from albumin and is also widely prescribed with a good safety profile [129].

## 8. Prospective Practice of Bilirubin Regulation in Obesity Treatment

There are several considerations in the prospective use of therapeutic approaches to modulate serum bilirubin levels in obese patients. First, it remains an open question whether the serum bilirubin level is a causal therapeutic target for obesity and CVDs, or rather a phenomenon driven by unmeasured confounding or reverse causation [130]. In this regard, large amounts of data have been obtained from several observational epidemiological studies, whereas there is a lack of controlled clinical trials offering the highest clinical evidence. Second, a recent large retrospective cross-sectional study on severely obese children showed that bilirubin is inversely associated only with some components of MetS, such as high blood pressure, high triglycerides, waist circumference, waist–height ratio, CRP levels, and HOMA-IR index; interestingly, no association between serum bilirubin and MetS or fatty liver was found [131]. This study showed that bilirubin is not protective in the presence of severe obesity (BMI > 95th percentile), whereas there is a greater number of published studies focused on the whole spectrum of individuals from overweight to obese. This raises the question of whether the role of bilirubin in obesity is more of a preventive nature or can also be therapeutic.

In contrast to most published studies, one study also reported that serum bilirubin concentrations were not negatively associated with inflammatory biomarkers or a protective lipid profile when conducting retrospective epidemiological investigations [132]; however, this study was conducted on all adults referred for routine medical check-ups, and most of the participants were indeed healthy with low CRP values and normal lipid profiles; thus, the protective effects of mildly increased serum bilirubin levels in overweight and obese individuals could have been masked.

The idea of using mildly elevated serum bilirubin levels in obesity treatment would require controlled clinical trials; however, since several drug candidates are already registered, this approach would involve drug repurposing with a known drug safety profile and potential adverse effects, whereas nutraceutical, diet, and physical activity approaches involve even more straightforward research processes. Moreover, it is important to mention that such interventions would be long term and must be individually tailored considering the severity of obesity and other CVD-related parameters.

Importantly, strong caution is required when using intervention approaches to increase bilirubin levels to prevent markedly elevated bilirubin levels, which might be toxic; thus, continuous monitoring of bilirubin levels in each individual must be employed, since the bilirubin levels largely depend on the individual genotype.

Our review article assesses the role of the serum bilirubin level as an additional clinical target that can be both modified and routinely measured, while considering that obesity is a multifactorial and complex disease with no single-hit druggable target; however, there is also a simple logic in the suggested approach. In obesity, chronic inflammation, increased oxidative stress, altered adipose tissue, and altered adipocyte metabolism occur; importantly, bilirubin is a strong endogenous antioxidant molecule with anti-inflammatory and adipocyte-modifying properties.

## 9. Conclusions

Overweight and obese adults have lower serum bilirubin values compared to normal-weight individuals. Since bilirubin is an endogenous molecule with antioxidant, anti-inflammatory, antithrombogenic, endocrine, and many other activities, the modulation of serum bilirubin levels represents a novel therapeutic approach. Mildly increased serum bilirubin levels will protect other organs and directly affect the adipose tissue and its adipokine secretion pattern. In our opinion, the modulation of serum bilirubin levels will be an effective adjunctive therapy for obesity that can improve several obesity-induced pathological conditions.

## Figures and Tables

**Table 1:** Adipokines and inflammatory markers that are altered in obese and overweight individuals and relationships with serum bilirubin levels.

Adipokines/Inflammatory Markers	Bilirubin Relationship	State	Reference
MCP-1	Inverse relationship	animal model (diet-induced obesity in mice)	Dong et al., 2014 [49]
TNF-α	Inverse relationship	overweight	Petelin et al., 2020 [42]
IL-6	Inverse relationship	overweight	Petelin et al., 2020 [42]
CRP	Inverse relationship	Overweight/obese/diabetic	Petelin et al., 2020 [42]; Yoshino et al., 2012 [5]; Melissas et al., 2006 [40]; Ohnaka et al., 2010 [41]
Adiponectin	Positive relationship	overweight	Petelin et al., 2020 [42]
Leptin	Inverse relationship	animal model (diet-induced obesity in mice)	Liu et al., 2015 [50]
Omentin-1	N/A	N/A	N/A
chemerin	Inverse relationship	cancer patients (colorectal carcinoma and hepatocellular carcinoma)	Feder et al., 2019 [51]
Resistin	Inverse relationship	overweight	Petelin et al., 2020 [42]
Visfatin	Inverse relationship	overweight	Petelin et al., 2020 [42]
Apelin	N/A	N/A	N/A
A-FABP	N/A	N/A	N/A
RBP-4	N/A	N/A	N/A

MCP-1 = monocyte chemoattractant protein 1; TNF-α = tumor necrosis factor α; IL-6 = interleukin 6; CRP = C-reactive protein; A-FABP = adipocyte fatty acid-binding protein; RBP-4 = retinol-binding protein 4; N/A = non-applicable, no literature data.
